# Impact of Substrate Preheating on Weld Quality, Microstructure, Corrosion Resistance, and Mechanical Properties in Gas Tungsten Arc Welding of UNS S32750 Super Duplex Stainless Steel

**DOI:** 10.3390/ma19020221

**Published:** 2026-01-06

**Authors:** Eli Jorge da Cruz Junior, Raul Henrique Ribeiro, Francisco Mateus Faria de Almeida Varasquim, Fábio Oliveira Carvalho, Luiz Fernando Frezzatti Santiago, Gabriela Pereira Lemos, Vicente Afonso Ventrella, Irene Calliari

**Affiliations:** 1 Department of Industry, São Paulo Federal Institute of Education, Science and Technology, Campus Itapetininga, Av. João Olímpio de Oliveira 1561, Itapetininga 18202-000, SP, Brazil; h.raul@aluno.ifsp.edu.br (R.H.R.); franciscomateus@ifsp.edu.br (F.M.F.d.A.V.); fabiocarvalho@ifsp.edu.br (F.O.C.); frezzatti@ifsp.edu.br (L.F.F.S.); 2 Department of Mechanical Engineering, Sao Paulo State University, Avenue Brasil 56, Ilha Solteira 15385-000, SP, Brazil; enggablemos@gmail.com (G.P.L.); vicente.ventrella@unesp.br (V.A.V.); 3 Department of Industrial Engineering, University of Padua, Via Gradenigo, 6/a, 35131 Padova, Italy; irene.calliari@unipd.it

**Keywords:** gas-shielded tungsten arc welding, super duplex stainless steel, heat treatment, volume fraction, corrosion resistance, microstructure

## Abstract

Super duplex stainless steels (SDSS) are materials known for their exceptional mechanical strength and high resistance to corrosion due to their dual- phase microstructure consisting of ferrite and austenite in roughly equal proportions. However, the Gas Tungsten Arc Welding (GTAW) process used to join SDSS often causes microstructural imbalances, mainly ferritic structures, or the formation of harmful intermetallic phases, which can weaken the material’ s desirable properties. This study examines the effect of substrate preheating on the microstructure, mechanical properties, and corrosion resistance of UNS S32750 SDSS welds produced by GTAW. Preheating the substrate was considered as a strategy to improve phase balance in the fusion zone by extending the time within the ferrite- to- austenite transformation temperature range, thus slowing the cooling rates. Four conditions were tested: welding at room temperature (RT) and preheating to 100 °C (T100), 200 °C (T200), and 300 °C (T300). Welding parameters remained constant. The fusion zone microstructure was analyzed using metallographic techniques, while mechanical properties were evaluated through microhardness tests. Corrosion resistance was assessed with potential dynamic polarization in a 3.5% NaCl solution. The results showed significant improvements in microstructural balance with higher preheating temperatures. The austenite volume fraction in the fusion zone increased from about 16% at RT to 42% at T 300. Mechanical testing indicated a decrease in microhardness from 341 HV at RT to 314 HV at T 300, reflecting the increased austenite content and its associated toughness. Corrosion tests demonstrated enhanced resistance under preheated conditions, with T 300 exhibiting the highest corrosion potential and the lowest corrosion current, nearing the performance of the base metal. These findings suggest that preheating is a practical, cost- effective method for optimizing the GTAW process for SDSS, eliminating the need for expensive filler materials and stabilizing the microstructure elements.

## 1. Introduction

Super duplex stainless steels (SDSS) are characterized by a dual-phase microstructure comprising nearly equal amounts of ferrite and austenite [1]. This unique structure offers an optimal balance of mechanical strength and corrosion resistance. Consequently, SDSS are widely utilized in demanding industries, including chemical processing, oil and gas exploration, petrochemicals, pulp and paper manufacturing, offshore platforms, and marine environments [2].

Welding super duplex stainless steel (SDSS) often results in a predominantly ferritic microstructure due to the rapid cooling rates typical of conventional methods such as Gas Tungsten Arc Welding (GTAW) [3]. This imbalance can reduce the material’s corrosion resistance and toughness, making it unsuitable for various industrial applications. Research shows that maintaining at least 25% austenite content is essential for most industrial uses, emphasizing the need for precise control of the phase balance during welding [4].

One key challenge in using SDSS is maintaining an appropriate phase ratio between ferrite and austenite during welding. Any imbalance can harm both the mechanical and corrosion properties of the material [5,6].

Traditional methods for achieving weld pool balance often involve adding austenite-stabilizing elements such as nickel or nitrogen [7,8,9,10,11,12,13,14,15]. While these approaches are effective, they can significantly raise production costs. A more affordable alternative is thermal treatments, which alter cooling rates and encourage the diffusion-controlled transformation of ferrite into austenite [16,17,18].

The literature presents several findings on the beneficial effects of heat treatments on the welding of SDSS (super duplex stainless steels). S. Saravanan et al. [19] reported an increase in the volume fraction of austenite after applying a post-weld heat treatment to pulsed Nd: YAG laser welds of SDSS. Köse and Topal [20] studied the effect of weld heat input and post-weld heat treatment (PWHT) on the microstructure, texture, surface, and mechanical properties of dissimilar (duplex stainless steel and austenitic stainless steel) laser beam welds. Pinheiro et al. [21] used a solubilization heat treatment on GTAW DSS welds and observed an increase in joint toughness, along with achieving a balanced microstructure. Ferrari et al. [22] also examined the impact of post-weld heat treatment on SDSS GTAW welds and similarly confirmed the beneficial effects on phase balance, corrosion resistance, and mechanical properties. A common feature across all these studies is that the treatment is performed after welding at temperatures above 1000 °C. In contrast, the effects of heat treatment before welding (substrate preheating), even for thin plates, as a means to promote phase balance and improve corrosion resistance and mechanical properties, are scarcely studied.

Preheating the substrate before welding has become a promising method to address microstructural imbalance during SDSS welding [23]. Preheating the substrate reduces cooling rates, allowing the fusion zone to stay longer in the ferrite-to-austenite transformation ranges. By increasing the material’s exposure to temperatures within this range, preheating promotes a more balanced microstructure without adding extra alloying elements [24]. Another advantage of the preheating method is that it operates at lower temperatures compared to Post-Weld Heat Treatment (PWHT). However, this method requires precise control of parameters to prevent the formation of unwanted secondary phases, which can damage the material’s mechanical and corrosion qualities [15,25].

Addressing issues related to the formation of intermetallic phases during welding and heat treatments is crucial. A common intermetallic that can form in super duplex stainless steels (SDSS) is chromium carbides. They form when carbon atoms cluster in the interstices at temperatures between 600 and 950 °C. Since they mainly form at the ferrite/austenite grain boundary during precipitation, chromium diffuses to create carbides, resulting in chromium-depleted zones next to the carbides [26]. This process is known as sensitization, and the affected region becomes vulnerable to intergranular corrosion.

Secondary phases, such as sigma or chi phases, can form within the temperature range of 700 to 950 °C, negatively impacting the material’s toughness and corrosion resistance. Research indicates that the sigma phase is particularly detrimental, as it adversely affects both weld toughness and corrosion resistance.

Another temperature range to avoid, especially during cooling, is between 400 and 500 °C. Prolonged exposure to this range can lead to the formation of the α’ phase, a chromium-rich structure that develops within the ferrite grain, which can cause embrittlement and reduce corrosion resistance [26,27]. Therefore, as mentioned, it is important to carefully control the involved temperatures and cooling rates to reduce the risk of these harmful phases [15].

The development of preheating techniques is becoming increasingly important due to the rising demand for standardized duplex stainless steels (SDSS) in extreme environments. In such situations, optimizing performance is critical for ensuring safety and durability. Research has shown that the cooling rates during welding not only influence the microstructural balance but also determine the mechanical properties and corrosion resistance of the final weld [4]. Therefore, it is essential to thoroughly understand the relationship between preheating parameters, cooling rates, and phase transformations to improve the application of gas tungsten arc welding (GTAW) in fabricating SDSS [25].

This study examines how substrate preheating affects the microstructure of UNS S32750 SDSS welded with Gas Tungsten Arc Welding (GTAW). The aim is to evaluate how preheating can improve the austenite content and achieve a proper phase balance for industrial uses while keeping the process cost-effective. Building on existing research, this work seeks to contribute to optimizing welding techniques for high-performance SDSS applications.

## 2. Materials and Methods

The material used in this study was UNS S32750 (TENAX, Rio de Janeiro, Brazil) super duplex stainless steel (SDSS) in the form of 1.5 mm thick sheets. The chemical composition of the base material (according to supplier) is provided in Table 1.

**Figure 1:**
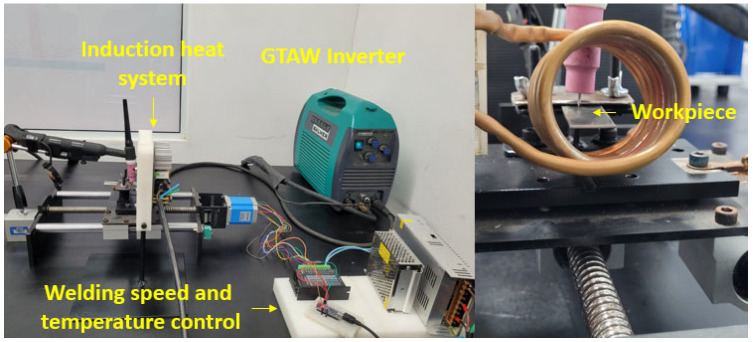
Welding setup.

Welding experiments were conducted using a Balmer MB 180 TIGP inverter (Balmer, João Pessoa, Brazil), connected to a linear guide and controlled by an Arduino system. To achieve preheating, an induction heating system was used, and temperature was monitored using a Flir thermographic camera (TG297) (FLIR, Wilsonville, OR, USA). The experimental setup kept consistent parameters throughout all tests. Figure 1 shows the welding setup.

Four conditions were analyzed: welding at room temperature (RT) and preheating at 100 °C (T100), 200 °C (T200), and 300 °C (T300). Bead-on-plate welding was performed using AWS EW7H2 tungsten electrodes (CARBOGRAFITE, Rio de Janeiro, Brazil) with a diameter of 1.6 mm, a 90° tip angle, and a 1 mm standoff distance from the plate. Argon shielding gas was used at a flow rate of 15 L/min.

Welding parameters were optimized to achieve a smooth, porosity-free weld surface, with a weld pool depth exceeding 50% of the sheet thickness. The chosen settings included a voltage of 29 V, a current of 45 A, and a welding speed of 500 mm/min.

After welding, samples were cut perpendicular to the weld and prepared for metallographic analysis. The microstructures were revealed using Beraha’s reagent and examined with an optical microscope (Carl Zeiss AxioCam ERc 5s—Jena, Germany. Volume fractions of ferrite and austenite were measured using the open-source image analysis software ImageJ v1.54r.

To evaluate the weldments’ tensile properties, samples were prepared according to ASTM E8/E8M-16ae1 standards [28] and tested on a universal testing machine EMIC DL 30.000 at room temperature. Tensile properties were assessed by setting the cross-head velocity to 2 mm/min to achieve a lower strain rate of 3.3 × 10^−4^ s^−1^.

Vickers microhardness tests were conducted on the clad layer, heat-affected zone (HAZ), and base metal using an EMCO TEST Duravision (Kuchl, Austria) with a load of 1 × 10^−3^ N.

To evaluate corrosion resistance, electrochemical tests were conducted in a 3.5% NaCl electrolyte solution at room temperature. The setup included an Ag/AgCl (3 M KCl) reference electrode and a platinum counter electrode. Potentiodynamic polarization curves were recorded using an AMETEK VersaSTAT 4 potentiostat at a scan rate of 1 mV/s. The data were then analyzed with Origin software v10.5.129.

## 3. Results

**Figure 2:**
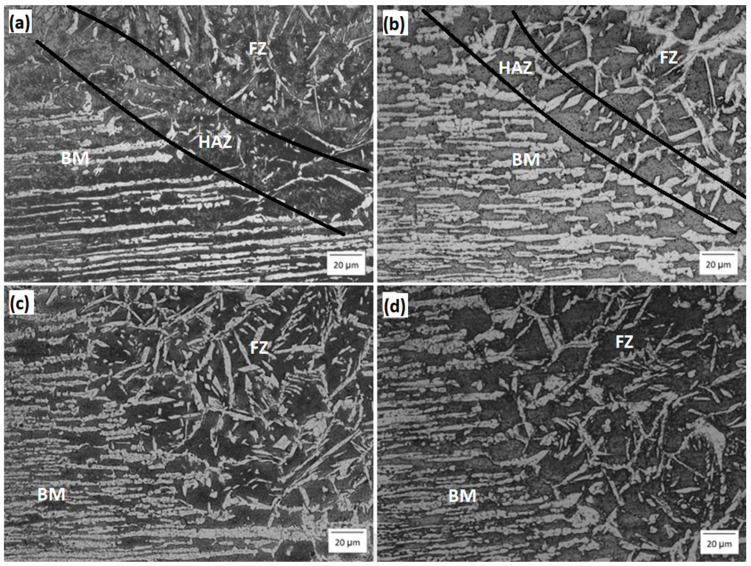
Micrograph transition region between base metal and fusion zone for (**a**) RT, (**b**) T100, (**c**) T200 and (**d**) T300.

The results from the welding experiments offered a comprehensive understanding of how different preheating conditions influence the microstructure, mechanical properties, and corrosion resistance of the fusion zones (FZ) in UNS S32750 SDSS welds. The microstructural analysis revealed clear changes in the distribution of austenite and ferrite phases across the four tested conditions: room temperature (RT) (a), T100 (b), T200 (c), and T300 (d). Figure 2 displays the micrographs of the FZ for each condition (with the HAZ indicated by a black line), highlighting a steady improvement in phase balance with increasing preheating temperatures.

In all observed conditions, two distinct areas of Gas Tungsten Arc Welding (GTAW) are identifiable: the base metal (BM) and the fusion zone (FZ). For the RT and T100 conditions, the Heat-Affected Zone (HAZ) is clearly defined. However, differentiating the HAZ from the FZ is difficult under other conditions. Additionally, micrographs show both epitaxial and competitive growth of columnar grains originating from the fusion line, with growth directed toward the area of heat extraction.

**Figure 3:**
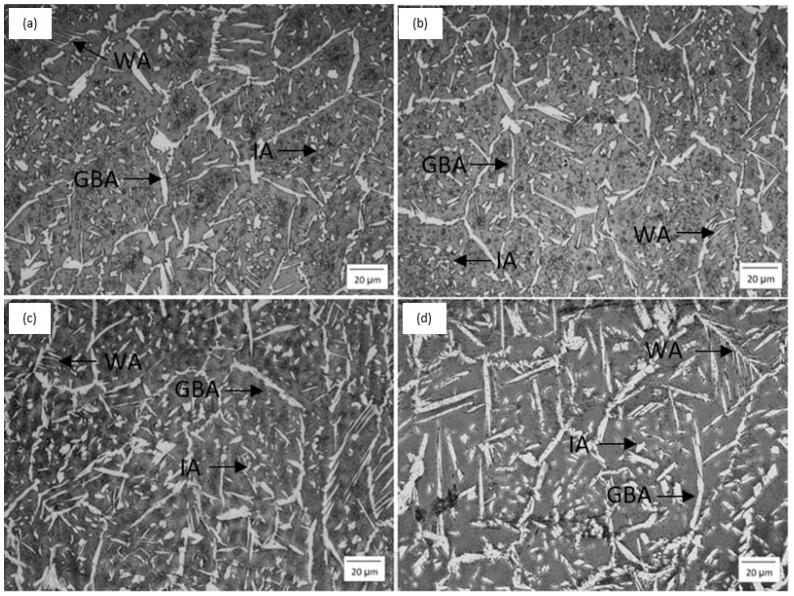
Micrographs of fusion zone (**a**) RT, (**b**) T100, (**c**) T200, (**d**) T300.

Figure 3 displays the FZ micrograph across all conditions. The variations in FZ microstructures are attributable to heat treatment (substrate preheating), despite the use of identical welding parameters.

After welding SDSS, different morphologies of austenite in the FZ microstructure are commonly observed, including grain boundary austenite (GBA), Widmanstätten austenite (WA), and intragranular austenite (IA). GBA forms at ferrite grain boundaries. WA nucleates from the grain boundary austenite and grows into the ferrite grains at an angle. IA develops within ferrite grains [7]. No significant changes in the morphology of these austenite types were seen across different conditions.

As the preheating temperature increases, more austenite forms. This occurs because higher preheating temperatures reduce the temperature difference between the fusion zone and the remaining base material, thereby slowing the cooling rate. The reduced cooling rate promotes the formation of austenite. It is well known that slower cooling rates favor austenite formation because they allow a longer period during which austenite grains emerge from δ-ferrite [21]. Despite the increased volume fraction of austenite, ferrite remained the dominant phase in all conditions, with a more pronounced imbalance at RT.

The volume fractions of austenite and ferrite were determined through image analysis and are shown in Table 2. The RT condition exhibited the lowest austenite fraction (15.8%), while preheating to 300 °C (T300) increased the austenite content to 42.4%. This increase underscores the effect of preheating on changing cooling rates and prolonging the material’s exposure to temperatures that promote the ferrite-to-austenite transformation.

The observed increases in the austenite fraction show that preheating helps overcome the cooling rate limitations of the GTAW process, encouraging the diffusional transformation of ferrite into austenite. However, despite these improvements, the ideal 50/50 phase balance was not achieved. This indicates that there is room for further optimization of preheating settings or the addition of more post-weld heat treatments.

Austenite forms via a solid-state diffusional transformation [24]. At high cooling rates, there is insufficient time for austenite to fully develop, as shown in the T100 condition and, more notably, in the TA condition. In contrast, the T200 and T300 conditions exhibit higher austenite volume fractions. This confirms that the preheating temperature affected the cooling rates, thereby allowing the temperature to remain within the range favorable for austenite formation for a longer period.

The phase balance in duplex stainless steel (DSS) is essential for maintaining its mechanical strength and corrosion resistance [3]. Research indicates that the weld bead should have a minimum austenite ratio of 25% to meet most industrial application requirements [2,4]. This underscores that effective heat treatment can increase the austenite volume fraction to levels that meet or exceed the minimum required for industrial use.

The results indicate that GTAW of SDSS can reduce costs by avoiding filler metals that promote austenite formation during welding.

The mechanical properties were evaluated through microhardness measurements in the fusion zone, with the results summarized in Table 3. The room temperature (RT) condition exhibited the highest hardness of 341 HV, consistent with its predominantly ferritic structure. As the austenite content increased with preheating temperature, the hardness values decreased. For example, the T300 condition exhibited a hardness of 314 HV, indicating the softer nature of austenite compared to ferrite.

The decline in hardness with increasing austenite content is consistent with the literature. Austenite improves toughness and ductility, which are important for applications requiring impact resistance. Although the decrease in hardness is notable, it does not compromise the material’s overall mechanical strength for industrial use.

**Figure 4:**
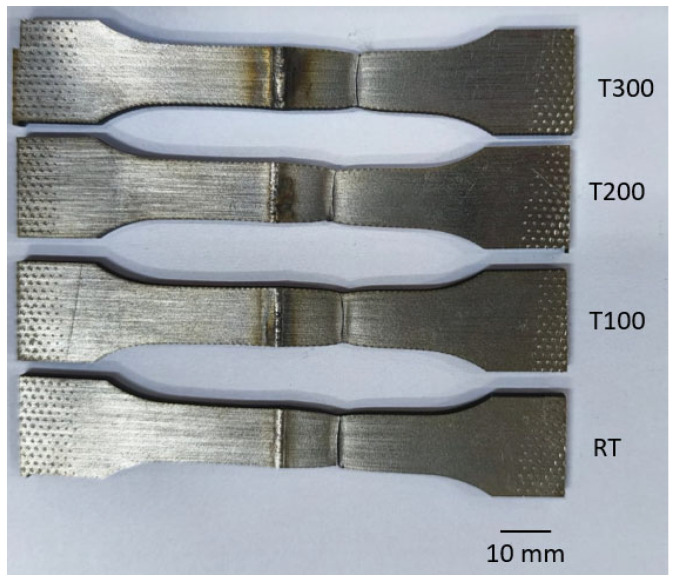
Tensile specimens.

Figure 4 displays a photograph of the specimens after testing. It is evident from the image that a fracture occurred in the base metal under all conditions, indicating that the weld beads were not embrittled and actually exhibited greater mechanical strength than the base metal. Although increasing the volume fraction of austenite generally tends to decrease the material’s mechanical strength [27], the weld bead’s strength remained higher than the base metal’s even under condition T300, where the austenite volume fraction was at its maximum. This observation is consistent with the hardness measurements.

**Figure 5:**
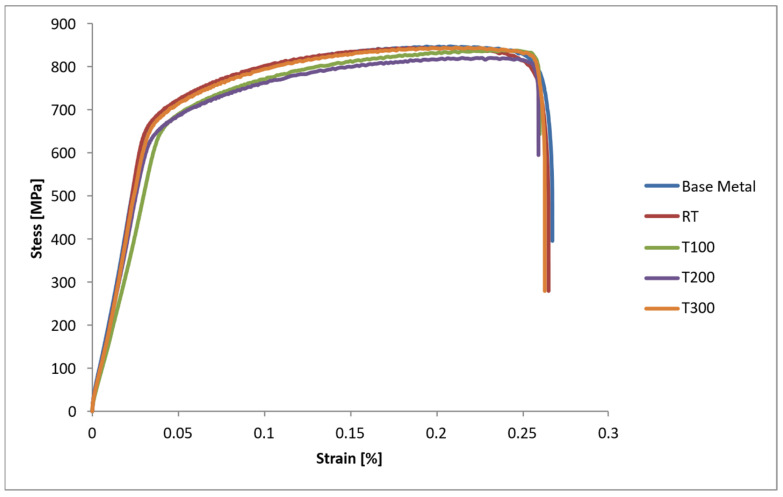
Stress–strain curves.

Figure 5 presents the stress–strain curves for all specimens. The curves clearly indicate that since fracture occurred in the base metal under all conditions, the ductility and ultimate tensile strength values matched those of the base metal. This indicates that the weld region did not experience embrittlement or a reduction in mechanical strength, even after heat treatment.

**Figure 6:**
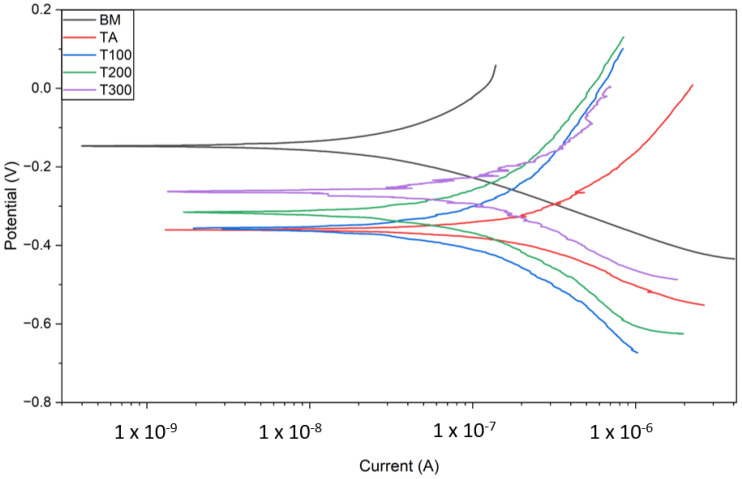
Tafel polarization curves.

Figure 6 shows the Tafel polarization curves for both the base metal and the welded samples. Higher corrosion potential values, which cause an upward shift in the Tafel curve, and lower corrosion current values, leading to a leftward shift, indicate enhanced corrosion resistance [29,30]. The higher potential seen in the base metal indicates a greater level of corrosion resistance. In contrast, the welded samples exhibited lower potentials, with the RT sample having the lowest potential and, therefore, the least corrosion resistance.

The corrosion resistance of SDSS is influenced by factors such as the α/γ ratio, grain size, and intermetallic phases. Typically, a higher fraction of austenite enhances corrosion resistance, resulting in a lower corrosion rate. This resistance is supported by the passivation layer [31]. Nitrogen, which enriches the austenite phase, strengthens this film by increasing its density, stability, and resistance to aggressive ions such as chlorides. A greater amount of austenite shifts the corrosion potential to more positive values and decreases the corrosion current [29].

The increase in corrosion resistance with higher preheating temperatures is attributable to increased austenite content, which improves the material’s pitting resistance. These findings confirm the dual benefit of preheating, as it enhances both the microstructure and corrosion resistance of super duplex stainless steel (SDSS) welds [30].

A comparison of the curves for the preheated samples and those welded from room temperature shows that the polarization curves shift upward toward more positive potentials and to the left toward lower currents. This change indicates a reduction in the corrosion rate, implying improved corrosion resistance. Table 4 shows the corrosion potentials and currents for all tested conditions.

The test results show that the RT and T100 samples exhibit the poorest corrosion resistance due to their high ferrite content. The potential dropped significantly from around −160 mV to −370 mV compared to the reference electrode, unlike the base metal. Conversely, the corrosion potentials of the T200 and T300 samples increased relative to the TA and T100 samples, with the T300 sample measuring about −270 mV.

The RT and T100 samples exhibited the highest corrosion currents, indicating lower corrosion resistance, as corrosion rates are directly proportional to these currents [32]. In contrast, the T300 sample had the lowest corrosion current among the welded samples, indicating the highest corrosion resistance.

This study demonstrates that preheating the substrate significantly enhances the phase balance, mechanical properties, and corrosion resistance of SDSS (Super Duplex Stainless Steel) welds made by Gas Tungsten Arc Welding (GTAW). Notably, unlike traditional Post Weld Heat Treatments (PWHT) performed at temperatures above 1000 °C, preheating achieves these improvements at a much lower temperature of around 300 °C. Micrographs and the testing of mechanical strength and corrosion resistance show that there was no embrittlement or decrease in corrosion resistance, indicating that preheating does not promote the formation of harmful secondary phases.

Although these improvements are significant, achieving a fully balanced microstructure is still a challenge, indicating that further tuning of the thermal parameters is needed. These findings lay a strong groundwork for using preheating as a cost-effective way to boost the performance of SDSS welds in industrial settings.

## 4. Conclusions

This study evaluated how substrate preheating affects the microstructure, mechanical properties, and corrosion resistance of UNS S32750 super duplex stainless steel (SDSS) welds produced by Gas Tungsten Arc Welding (GTAW). The main conclusions are summarized as follows:

1. Preheating the substrate effectively reduced the phase imbalance caused by the GTAW process, significantly increasing the volume fraction of austenite in the fusion zone. The austenite fraction improved from 15.8% of the sample welded starting from room temperature to 42.4% in the heated condition.
2. As the austenite content increased with higher preheating temperatures, the microhardness values in the fusion zone decreased. The room temperature (RT) condition exhibited the highest hardness at 341 HV, while the T300 condition demonstrated improved toughness with a hardness of 314 HV, indicating enhanced ductility.
3. Preheating improved the corrosion resistance of the fusion zones. The condition identified as T300 displayed the lowest corrosion current and the highest corrosion potential, indicating a superior resistance to localized corrosion compared to the room temperature (RT) condition.
4. While the T300 condition did not achieve a completely balanced microstructure, the improvements in phase balance, mechanical properties, and corrosion resistance make substrate preheating a practical and cost-effective strategy for the industrial application of SDSS welds.
5. Unlike traditional Post Weld Heat Treatments (PWHT) that need temperatures over 1000 °C, preheating achieves similar benefits at around 300 °C. Importantly, microstructural analysis, mechanical tests, and corrosion tests reveal no evidence of embrittlement or reduced corrosion resistance, confirming that preheating does not lead to harmful secondary-phase formation.
6. This study highlights the potential of substrate preheating to enhance the efficiency and effectiveness of welding processes for SDSS, making it a valuable method for demanding industrial applications.

## Figures and Tables

**Table 1:** Chemical composition of UNS S32750 (wt-%).

Cr	Ni	Mo	Mn	Si	N	Cu	P	C
25.61	6.97	3.84	0.63	0.29	0.27	0.15	0.02	0.018

**Table 2:** Volume Fractions.

	Austenite [%]	Ferrite [%]	SD
BM	50.1	49.9	0.2
RT	15.8	84.2	2.1
T100	16.5	83.5	1.9
T200	25.8	74.2	1.5
T300	42.4	57.6	2.3

**Table 3:** Microhardness values.

	Hardness [HV]
BM	302 ± 2
RT	341 ± 2
T100	342 ± 3
T200	327 ± 2
T300	314 ± 1

**Table 4:** Corrosion potentials and currents.

	Corrosion Potential [v]	Corrosion Current [a]
MB	−0.16	3.2 × 10^−9^
RT	−0.38	3.4 × 10^−8^
T100	−0.37	2.5 × 10^−8^
T200	−0.31	9.9 × 10^−9^
T300	−0.27	6.3 × 10^−9^

## Data Availability

The original contributions presented in this study are included in the article. Further inquiries can be directed to the corresponding author.
